# Predictive value of preoperative serum CCL2, CCL18, and VEGF for the patients with gastric cancer

**DOI:** 10.1186/1472-6890-13-15

**Published:** 2013-05-22

**Authors:** Jianghong Wu, Xiaowen Liu, Yanong Wang

**Affiliations:** 1 Department of Gastric Cancer and Soft Tissue Sarcoma, Fudan University Shanghai Cancer Center, 270 Dong An Road, Shanghai 200032, People’s Republic of China; 2 Department of Oncology, Shanghai Medical College, Fudan University, Shanghai 200032, China

**Keywords:** Gastric cancer, Diagnosis, Biological markers

## Abstract

**Background:**

To investigate the expression of chemokine ligand 2 (CCL2), chemokine ligand 18 (CCL18), and vascular endothelial growth factor (VEGF) in peripheral blood of patients with gastric cancer and their correlation with presence of malignancy and disease progression.

**Methods:**

Sixty patients with pathological proved gastric cancer were prospectively included into study. The levels of CCL2, CCL18, and VEGF in peripheral blood were examined by enzyme-linked immunosorbentassay (ELISA). Peripheral blood from 20 healthy people was examined as control.

**Results:**

The preoperative serum levels of CCL2, CCL18 and VEGF in gastric cancer patients were significantly higher than that of controls (*P* <0.001, *P* <0.001, and *P* <0.001, respectively). ROC curve analysis showed that with a cut-off value of ≥1272.8, the VEGF*CCL2 predicted the presence of gastric cancer with 83% sensitivity and 80% specificity. Preoperative serum CCL2 was significantly correlated to N stage (*P* =0.040); CCL18 associated with N stage (*P* =0.002), and TNM stage (*P* =0.002); VEGF correlated to T stage (*P* =0.000), N stage (*P* =0.015), and TNM stage (*P* =0.000).

**Conclusion:**

Preoperative serum levels of CCL2 and VEGF could play a crucial role in predicting the presence and progression of gastric cancer.

## Background

Although the incidence of gastric cancer has been substantially declining for several decades, it was still the fourth most common cancer and the second most frequent cause of cancer death [1,2]. The high death rate was related to the difficulty of detecting gastric cancer at an early stage. Some serum tumor markers such as AFP, CEA, CA19-9, CA72-4, and CA50 were currently the best clinical markers for gastric cancer. However, they were no good diagnostic tumor makers in early stage gastric cancer. Therefore, it was important to develop new markers for detecting gastric cancer.

Chemokines were a kind of low-molecular weight cytokines, which were implicated in many biological processes, such as migration of leukocytes, angiogenesis, and tumor growth. The CCL chemokines represented the largest family of chemokines. They could attract monocytes, macrophages, T cells, B cells, eosinophils, dendritic cells, mast cells and natural killer cells [3]. CCL2 was a 76-amino acid protein that was originally purified and cloned from human gliomas and myelomonocytic cells in 1989 [4]. CCL2 was the first chemokine discovered in the C-C subfamily of chemokines and was produced by a variety of cells. Some studies have implicated that CCL2 was an active participant in the tumor microenvironment [5,6]. CCL18 was a newly defined member of C-C subgroup of chemokines. It played an important role in inflammation and generation of the immune response. However, the involvement of CCL18 in cancer was not clear.

Vascular endothelial growth factor (VEGF) was firstly isolated from the culture supernatant and the ascites of rodent tumors a potent vascular permeability factor [7]. It was thought to be one of the most important factors promoting vascularization [8]. It played a role in both physical and malignant conditions [9]. Previous studies reported that serum VEGF level was high in several cancers, such as breast cancer and colon cancer [10,11].

The aim of this study was to investigate the relationship between the preoperative serum levels of CCL2, CCL18, and VEGF, and the clinicopathological factors in patients with gastric cancer.

## Methods

### Study population

From July 2005 to December 2005, 60 patients who underwent curative gastrectomy at department of Gastric Cancer and Soft Tissue Sarcoma in Shanghai Cancer Hospital were included into this study. All patients were confirmed as gastric cancer by preoperative biopsy. No patients received neoadjuvant treatment or preoperative blood transfusion. Additionally, 20 normal persons were designated as the control. Staging was performed according to the American Joint Committee on Cancer (AJCC) TNM Staging Classification for Carcinoma of the Stomach (6th edition, 2002). Data were retrieved from patients’ operative and pathological reports. The written informed consent had been obtained from all the patients, and this study was approved by the Ethical Committee of Shanghai Cancer Center of Fudan University.

### Blood samples

Blood samples were obtained by peripheral venous puncture on the day before operation. After clotting and within an hour of collection, the blood samples were centrifuged at 3000 g for 5 min and serum aliquots were stored at -80°C until analysis.

### ELISA

The measurement of CCL2, CCL18, and VEGF was performed using Quantikine human CCL2, CCL18, and VEGF sandwich enzyme-linked immunosorbent assay (ELISA) kits (Genzyme Corporation, USA) according to the manufacturer’s instructions. All assays were duplicated.

### Statistical analysis

Continuous variables and categorical variables were expressed as mean ± 1 standard deviation and percentages, respectively. The Kolmogorov-Smirnov test was used to assess whether the continuous variables conformed to a Gaussian pattern. Comparisons of continuous variables between independent groups were performed with Student’s unpaired *t*-test or Mann–Whitney non-parametric test. Comparisons of continuous variables between related groups were performed with Student’s paired *t*-test and non-parametric Wilcoxon’s signed-rank test. Linear correlations were assessed by calculating Pearson’s correlation coefficient or the non-parametric Spearman’s rho. The accepted level of significance was *P* <0.05. Statistical analyses and graphics were performed with the SPSS 13.0 statistical package (SPSS, Inc., Chicago, IL).

## Results

### Clinicopathological characteristics

There were 14 males and 6 females (2.33:1) with a mean age of 56 years in normal persons. There were 41 males and 19 females (2.16:1) with a mean age of 55 years. There was 7 (11.7%) early gastric cancers and 53 (88.3%) advanced gastric cancers. According to histological type, well-differentiated tumors were observed in 1 (1.7%) patients, moderately-differentiated in 18 (30.0%) patients, and poorly-differentiated tumors in remaining 41 (68.3%) patients. Lymph node metastasis was observed in 40 patients, the metastasis rate was 66.7%. The distribution of pathological stage was as follows: 11 (18.3%) patients belonged to stage I, 9 (15.1%) II, 23 (38.3%) III, 17 (28.3%) IV.

### Serum CCL12, CCL18, and VEGF values

The serum values of CCL2, CCL18, and VEGF in normal persons were 15.95 ± 1.15 (pg/ml), 42.46 ± 13.97 (pg/ml), and 59.02 ± 6.28 (pg/ml), respectively. The serum values of CCL2, CCL18, and VEGF in gastric cancers were 25.05 ± 1.22 (pg/ml), 115.94 ± 22.56 (pg/ml), and 132.92 ± 10.50 (pg/ml), respectively. The levels were significantly higher in patients than that of control groups (Table 1).

**Table 1:** Comparisons of serum CCL2, CCL18, and VEGF levels between gastric cancer patients and control groups

	**N**	**Mean (pg/ml)**	**Std. deviation**	**P**
CCL2				< 0.001
Patients	60	25.05	1.22	
Controls	20	15.95	1.15	
CCL18				< 0.001
Patients	60	115.94	22.56	
Controls	20	42.46	13.97	
VEGF				< 0.001
Patients	60	132.92	10.50	
Controls	20	59.02	6.28	

### Presence of malignancy

Serum VEGF and CCL2 levels were significant independent predictors for the presence of gastric cancer. The optimal predictive model (*χ*^2^ =41.470, df =3, N =80, Negelkerke R^2^ =0.599, *P* <0.001) predicted presence of malignancy with 93.3% sensitivity and 60.0% specificity. With a serum VEGF levels cut-off values ≥ 68.2 pg/ml, the sensitivity and specificity of VEGF to distinguish patients from controls were 83% and 60%, respectively. In contrast, with a serum CCL2 levels cut-off values ≥ 16.6 pg/ml, the sensitivity and specificity of CCL2 to distinguish patients from controls were 78% and 60%, respectively.

**Figure 1:**
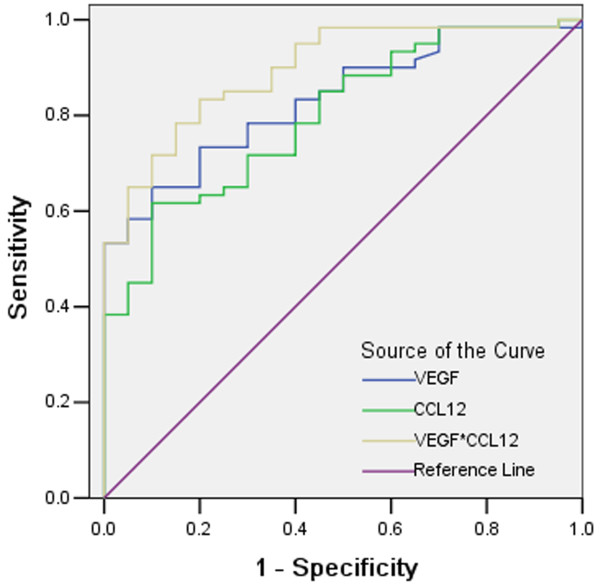
**Combined ROC curve of serum CCL2 (area under the curve 0.79, *P*** **< 0.001), VEGF (area under the curve 0.83, *P*** < 0.001) and CCL2*VEGF cut-off values for the presence of gastric cancer. The ration value was the predictor of gastric cancer with the largest area under the curve (0.90, *P* < 0.001).

With a cut-off value of ≥1272.8, the VEGF*CCL2 predicted the presence of gastric cancer with 83% sensitivity and 80% specificity (Table 2). The concentrations product yielded the largest area under the ROC curve (0.895, *P* <0.001) (Figure 1).

**Table 2:** Cut-off values, sensitivity and specificity of VEGF*CCL2 for distinguishing gastric cancer patients from controls

**Cut-off/upper than or equal (+)**	**Sensitivity (%)**	**Specificity (%)**
VEGF*CCL2		
1205.6	85	70
1212.6	85	75
1242.6	83	75
**1272.8**	**83**	**80**
1291.4	82	80
1332.3	80	80

In bold, the reference cut-off value that combines optimal sensitivity and specificity for clinical use.

### Correlation of preoperative serum markers and clinicopathological features

Preoperative serum CCL2 was significantly correlated to age (r =0.433, *P* =0.001) and N stage (r = -0.266, *P* =0.040). Preoperative serum CCL18 was significantly associated with histology type (r = -0.296, *P* =0.022), N stage (r = -0.384, *P* =0.002), and TNM stage (r = -0.386, *P* =0.002). Preoperative serum VEGF was significantly correlated to T stage (r =0.598, *P* =0.000) and N stage (r = 0.312, *P* =0.015), and TNM stage (r =0.531, *P* =0.000). There were no statistically significant correlation of CCL2, CCL18, and VEGF to gender, vascular invasion, and nervous invasion (Table 3).

**Table 3:** The relation between preoperative serum VEGF, CCL12, CCL18 and clinicopathological parameters in gastric cancer patients

**Parameters**	**VEGF**		**CCL2**		**CCL18**	
	**r**	***P***	**r**	***P***	**r**	***P***
T	0.598	0.000	−0.065	0.623	−0.092	0.485
N	0.312	0.015	−0.266	0.040	−0.384	0.002
M	0.166	0.204	0.046	0.724	−0.220	0.091
TNM	0.531	0.000	−0.118	0.370	−0.386	0.002
Histological type	−0.115	0.382	0.164	0.211	−0.296	0.022
Venous invasion	−0.032	0.807	0.089	0.499	−0.112	0.393
Neuron invasion	0.061	0.644	−0.006	0.962	−0.025	0.848
Gender	0.029	0.826	0.125	0.341	0.088	0.504
Age	0.123	0.348	0.433	0.001	−0.084	0.525

*r* Correlation coefficients (r).

## Discussion

Poor survival of gastric cancer was largely due to late-stage diagnosis. Late-stage diagnosis can be attributed to the fact that the disease was relatively “asymptomatic” in its early stage and nonspecific complaints in its late-stage. Therefore, it was necessary to find out some markers for early diagnosis of gastric cancer. Thus, we measured the concentration of CCL2, CCL18, and VEGF in a series of 60 serum samples from gastric cancer. Additionally, a series of 20 serum samples from healthy people was selected as controls. Moreover, we investigated the relationship between the levels of these markers and the clinicopathological factors. To our knowledge, no published studies have simultaneously studied the diagnostic value of serum CCL2, CCL18, and VEGF in gastric cancer patients.

In the current study, we found that the serum levels of CCL2, CCL18, and VEGF markedly increased in gastric cancer group compared to control group. It was generally accepted that CCL2 was a potent proinflammatory mediator produced by several cells including monocytes [12]. Additionally, tumor cells or peritumoral components can produce cytokines [13]. CCL2 has been investigated in serum of patients with some tumors like breast cancer, ovarian tumor, and gastric cancer [13-15]. Tonouchi et al. [15] reported that the serum concentration of CCL2 in patients with carcinoma was significantly lower than that in controls, which was not consistent with the current study. In this study, we found that the serum levels of CCL2 markedly increased in gastric cancer group compared to control group. The exact mechanism resulting in this contradiction was not clear. It was possible that the decreased serum level of CCL2 may reflect increased local consumption in tumor [15]. However, others have shown statistically significant increases in serum CCL2 levels in patients compared with healthy controls [13,14]. CCL18 played a role in the homing of T and B cells participating in the immune response. It was reported that CCL18 was correlated to the autoimmune disease. However, the reports about CC18 in cancer were rare. In this study, we found that CCL18 markedly increased in gastric cancer group compared to control group. However, it was not independent predictor for presence of gastric cancer. It was well known that vascular endothelial growth factor (VEGF) family played an important role in vasculogenesis and angiogenesis. Among the VEGF family members, VEGF-A played an essential role in angiogenesis. However, the significant value of serum levels of other VEGF members (VEGF-C and VEGF-d) has been reported [16-18]. However, previous studies on serum VEGF-C and VEGF-D in gastric cancer patients have presented with contradictory results. Wang et al. [16] reported that higher serum levels significantly associated with the presence of malignancy and lymph node metastasis. To the contrary, AI-Moundhri et al. [17] did not showed significant differences of serum VEGF-C between patients and control groups, and higher VEGF-D in patients. In review of inconsistent reports, we conducted logistic regression analysis, and found that serum VEGF was an independent predictor of the presence of gastric cancer. Furthermore, we performed the ROC curve to investigate the predictive value of gastric cancer, and results showed that VEGF*CCL2 could provide higher accuracy for distinguishing gastric cancer patients from controls than alone VEGF or CCL.

The correlation of CCL2, CCL18, and VEGF with clinicopathological variables has been elucidated in various tumors. Tonouchi H et al. reported that the concentration of serum CCL2 in gastric cancer patients decreased in accordance with disease progression [15]. Zohny SF et al. reported that serum CCL18 was significantly increased in epithelial ovarian cancer with early stages compared to those with late stages [19]. In this study, we found the similar results. It was suggested that the consumption of CCL2 and CCL18 increased and production decreased in accordance with disease progression.

## Conclusions

CCL2 played an important role in immune response to tumor. Together with VEGF, they were useful biomarkers in predicting gastric cancer.

## Competing interests

The authors declare that they have no competing interests.

## Authors’ contributions

Conceived and designed the experiments: JW XL YW. Performed the experiments: JW XL. Analyzed the data: JW XL YW. Wrote the paper: JW XL. All authors read and approved the final manuscript.

## Pre-publication history

The pre-publication history for this paper can be accessed here:

http://www.biomedcentral.com/1472-6890/13/15/prepub
